# Characteristics of Initial Prescription Episodes and Likelihood of Long-Term Opioid Use — United States, 2006–2015

**DOI:** 10.15585/mmwr.mm6610a1

**Published:** 2017-03-17

**Authors:** Anuj Shah, Corey J. Hayes, Bradley C. Martin

**Affiliations:** ^1^Division of Pharmaceutical Evaluation and Policy, College of Pharmacy, University of Arkansas for Medical Sciences; ^2^Division of Health Services Research, College of Medicine, University of Arkansas for Medical Services.

Because long-term opioid use often begins with treatment of acute pain (*1*), in March 2016, the CDC Guideline for Prescribing Opioids for Chronic Pain included recommendations for the duration of opioid therapy for acute pain and the type of opioid to select when therapy is initiated (*2*). However, data quantifying the transition from acute to chronic opioid use are lacking. Patient records from the IMS Lifelink+ database were analyzed to characterize the first episode of opioid use among commercially insured, opioid-naïve, cancer-free adults and quantify the increase in probability of long-term use of opioids with each additional day supplied, day of therapy, or incremental increase in cumulative dose. The largest increments in probability of continued use were observed after the fifth and thirty-first days on therapy; the second prescription; 700 morphine milligram equivalents cumulative dose; and first prescriptions with 10- and 30-day supplies. By providing quantitative evidence on risk for long-term use based on initial prescribing characteristics, these findings might inform opioid prescribing practices.

A random 10% sample of patient records during 2006–2015 was drawn from the IMS Lifelink+ database, which includes commercial health plan information from a large number of managed care plans and is representative of the U.S. commercially insured population (*3*). The data are provided in a deidentified format and the institutional review board at the authors’ institution deemed the study was not human subject research. Records were selected of patients aged ≥18 years who had at least one opioid prescription during June 1, 2006–September 1, 2015, and ≥6 months of continuous enrollment without an opioid prescription before their first opioid prescription. Patients excluded were those who had any cancer (other than nonmelanoma skin cancer) or a substance abuse disorder diagnosis in the 6 months preceding their first opioid prescription, or whose first prescription was for any buprenorphine formulation indicated for treatment of substance abuse.

Patients were followed from the date of their first prescription until loss of enrollment, study end date, or discontinuation of opioids, which was defined as ≥180 days without opioid use. The duration of use and number of prescriptions and cumulative dose (expressed in morphine milligram equivalents*) for the first episode of opioid use (defined as continuous use of opioids with a gap of no greater than 30 days) were calculated. The number of days’ supply and average daily dose in morphine milligram equivalents for the first prescription were also calculated. The first opioid prescription was categorized into six mutually exclusive categories: long-acting; oxycodone short-acting; hydrocodone short-acting; other Schedule II short-acting; Schedule III–IV and nalbuphine; and tramadol.^†^

The Kaplan-Meier statistic was used to estimate median time to discontinuation of opioid use; probability of continued opioid use at 1 year and 3 years for different treatment duration thresholds (daily for 1–40 days and weekly for 1–26 weeks); number of prescriptions (1–15); and cumulative dose of the first episode of opioid use (50–2000 morphine milligram equivalents). Similarly, the relationship between the number of days’ supply, choice of first opioid prescription, and probability of continued opioid use at 1 and 3 years was also examined. Sensitivity analyses were conducted by modifying the discontinuation definition from ≥180 opioid-free days to ≥90 opioid-free days, changing the allowable gap in the first episode of opioid use from 30 days to 7 days, and excluding patients whose average daily dose of the first prescription exceeded 90 morphine milligram equivalents.

A total of 1,294,247 patients met the inclusion criteria, including 33,548 (2.6%) who continued opioid therapy for ≥1 year. Patients who continued opioid therapy for ≥1 year were more likely to be older, female, have a pain diagnosis before opioid initiation, initiated on higher doses of opioids, and publically or self-insured, compared with patients who discontinued opioid use in <365 days (Table). Among persons prescribed at least 1 day of opioids, the probability of continued opioid use at 1 year was 6.0% and at 3 years was 2.9% (supplemental figure 1; https://stacks.cdc.gov/view/cdc/44182) (supplemental figure 2; https://stacks.cdc.gov/view/cdc/44550) with a median time to discontinuation of 7 days (supplemental figure 3; https://stacks.cdc.gov/view/cdc/44551). Approximately 70% of patients have an initial duration of opioids of ≤7 days and 7.3% were initially prescribed opioids for ≥31 days. The largest incremental increases in the probability of continued opioid pain reliever use were observed when the first prescription supply exceeded 10 or 30 days (Figure 1), when a patient received a third prescription (Figure 2), or when the cumulative dose was ≥700 morphine milligram equivalents (supplemental figure 4; https://stacks.cdc.gov/view/cdc/44552). Substantial increases in probabilities of continued opioid use occurred when the initial duration reached 6 and 31 days (supplemental figure 2; https://stacks.cdc.gov/view/cdc/44550); the findings of the sensitivity analyses were similar (supplemental figures 5–10; https://stacks.cdc.gov/view/cdc/44183).

**TABLE:** Characteristics of incident opioid users and patients who continued opioid use for ≥365 days (1 year) and ≥1,095 days (3 years) — United States, 2006–2015

Characteristic	All incident opioid users		Patients who continued opioid therapy for ≥365 days		Patients who continued opioid therapy for ≥1,095 days	
	(N = 1,294,247)		(n = 33,548)		(n = 6,441)	
	Mean (SD)	95% CI	Mean (SD)	95% CI	Mean (SD)	95% CI
Duration of first episode of opioid use	14.81 (65.00)	14.70–14.92	183.28 (343.27)	179.61–186.96	362.40 (593.26)	347.91–376.90
Enrollment duration (yrs)	2.48 (2.04)	2.47–2.48	3.30 (1.83)	2.47–2.48	4.98 (1.48)	4.94–5.02
Age (yrs)	44.52 (14.56)	44.50–44.54	49.58 (13.45)	49.44–49.72	50.52 (12.68)	50.21–50.83
Female	**No. (%)**	**95% CI**	**No. (%)**	**95% CI**	**No. (%)**	**95% CI**
	698,950 (54.00)	53.92–54.09	18,768 (55.94)	55.41–56.47	3,500 (54.34)	53.12–55.55
**Treatment indication**						
Back pain	226,681 (17.51)	17.45–17.58	10,396 (30.99)	30.50–31.49	2,137 (33.18)	32.04–34.34
Neck pain	90,352 (6.98)	6.94–7.03	3,824 (11.40)	11.06–11.74	775 (12.03)	11.26–12.85
Head pain	30,123 (2.33)	2.30–2.35	1,495 (4.46)	4.24–4.68	306 (4.75)	4.26–5.30
Joint pain	389,700 (30.11)	30.03–30.19	14,862 (44.30)	43.77–44.83	2,968 (46.08)	44.87–47.30
**Patient region**						
South	476,565 (36.74)	36.64–36.83	13,437 (40.05)	39.53–40.53	2,449 (38.02)	36.84–39.21
Midwest	376,520 (29.09)	29.01–29.17	9,566 (28.51)	28.03–29.00	1,973 (30.63)	29.52–31.77
East	279,595 (21.60)	21.53–21.67	6,153 (18.34)	17.93–18.76	1,234 (19.16)	18.22–20.14
West	142,698 (11.03)	10.97–11.08	3,640 (10.85)	10.52–11.19	574 (8.91)	8.24–9.63
Missing/Other	19,869 (1.54)	1.51–1.56	752 (2.24)	2.09–2.41	211 (3.28)	2.87–3.74
**Payer type**						
Commercial	866,815 (66.97)	66.89–67.06	20,920 (62.36)	61.84–62.88	3,910 (60.70)	38.11–40.49
Medicaid/State CHIP	14,855 (1.15)	1.13–1.17	864 (2.58)	2.42–2.76	154 (2.39)	2.05–2.79
Medicare	16,951 (1.31)	1.29–1.33	1,160 (3.46)	3.27–3.66	257 (3.96)	3.52–4.48
Self-insured	387,122 (29.91)	29.83–29.99	10,471 (31.21)	30.72–31.71	2,089 (32.43)	31.30–33.59
RX only/Unknown	8,504 (0.66)	0.64–0.67	130 (0.39)	0.33–0.46	32 (0.50)	0.35–0.70
**Prescription characteristic**						
First prescription ≥90 MME*	89,438 (6.91)	6.87–6.95	2,613 (7.79)	7.51–8.08	545 (8.46)	7.81–9.17
First prescription ≥120 MME*	22,895 (1.77)	1.75–1.79	1,075 (3.20)	3.02–3.40	244 (3.79)	3.35–4.28
First long-acting opioid prescription^†^	6,588 (0.51)	0.50–0.52	905 (2.70)	2.53–2.88	226 (3.51)	3.09–3.99

**Abbreviations:** CHIP = Children’s Health Insurance Plan; CI = confidence interval; MME = morphine milligram equivalents; RX = prescription; SD = standard deviation.

*Average daily dose was calculated as total strength of the prescription expressed in MME divided by the days’ supply of the first prescription. If a patient had multiple prescriptions on the first day, the daily dose in MME for all the prescriptions on the index date were summed and divided by the days’ supply of the longest lasting prescription.

^†^ The first prescription was categorized into six mutually exclusive categories and, in case of multiple prescriptions, on the index date using the following hierarchy to assign category: 1) long-acting; 2) other Schedule II short-acting; 3) Oxycodone short-acting; 4) Hydrocodone short-acting; 5) Schedule III-IV and Nalbuphine; or 6) tramadol.

**FIGURE 1:**
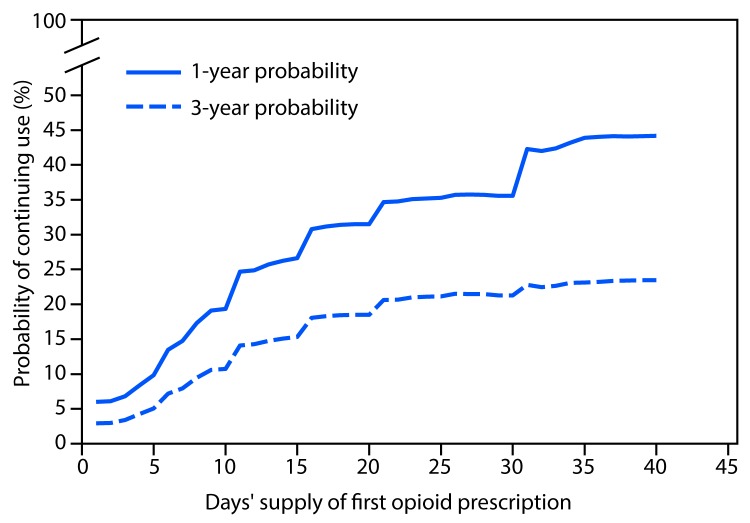
One- and 3-year probabilities of continued opioid use among opioid-naïve patients, by number of days’ supply* of the first opioid prescription — United States, 2006–2015 * Days’ supply of the first prescription is expressed in days (1–40) in 1-day increments. If a patient had multiple prescriptions on the first day, the prescription with the longest days’ supply was considered the first prescription.

**FIGURE 2:**
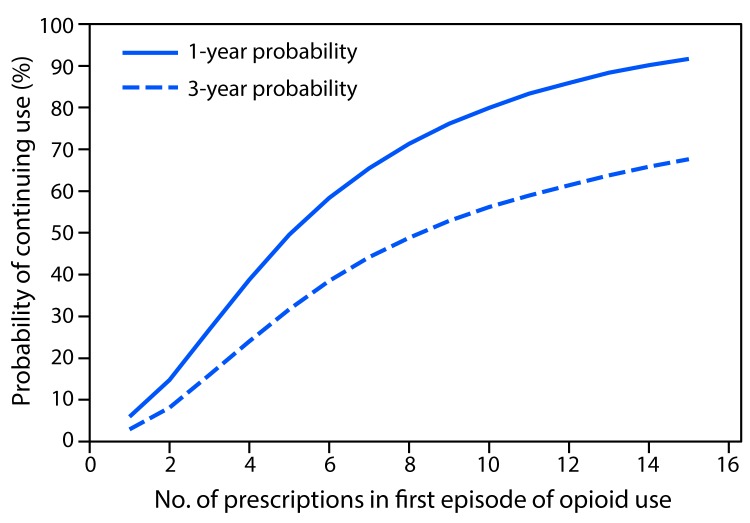
One- and 3-year probabilities of continued opioid use among opioid-naïve patients, by number of prescriptions* in the first episode of opioid use — United States, 2006–2015 * Number of prescriptions is expressed as 1–15, in increments of one prescription.

The highest probabilities of continued opioid use at 1 and 3 years were observed among patients who initiated treatment with a long-acting opioid (27.3% at 1 year; 20.5% at 3 years), followed by those whose initial treatment was with tramadol (13.7% at 1 year; 6.8% at 3 years) or a Schedule II short-acting opioid other than hydrocodone or oxycodone (8.9% at 1 year; 5.3% at 3 years) (supplemental table; https://stacks.cdc.gov/view/cdc/44181). The probabilities of continued opioid use at 1 and 3 years for persons starting on hydrocodone short acting (5.1% at 1 year; 2.4% at 3 years), oxycodone short-acting (4.7% at 1 year; 2.3% at 3 years), or Schedule III–IV (5.0% at 1 year; 2.2% at 3 years) opioids were similar (supplemental table; https://stacks.cdc.gov/view/cdc/44181).

## Discussion

The probability of long-term opioid use increases most sharply in the first days of therapy, particularly after 5 days or 1 month of opioids have been prescribed, and levels off after approximately 12 weeks of therapy. The rate of long-term use was relatively low (6.0% on opioids 1 year later) for persons with at least 1 day of opioid therapy, but increased to 13.5% for persons whose first episode of use was for ≥8 days and to 29.9% when the first episode of use was for ≥31 days. Although ≥31 days of initial opioid prescriptions are not common, approximately 7% do exceed a 1-month supply. Discussions with patients about the long-term use of opioids to manage pain should occur early in the opioid prescribing process, perhaps as early as the first refill, because approximately 1 in 7 persons who received a refill or had a second opioid prescription authorized were on opioids 1 year later. As expected, patients initiated on long-acting opioids had the highest probabilities of long-term use. However, the finding that patients initiated with tramadol had the next highest probability of long-term use was unexpected; because of tramadol’s minimal affinity for the *μ*-opioid receptor, it is deemed a relatively safe opioid agonist with lower abuse potential than other opioids (*4*). However, a report by the Substance Abuse and Mental Health Services Administration determined that emergency department visits associated with tramadol-related adverse events increased by 145% during 2005–2011 (*5*). Long-term data on tramadol for pain management are sparse, with only one trial exceeding 12 weeks in duration (*6*). Despite this, among patients initiated with tramadol, >64% of patients who continued opioid use beyond 1 year were still on tramadol, suggesting that tramadol might be prescribed intentionally for chronic pain management. A 2016 study in Oregon (*7*), which did not include tramadol (a predictor of long-term use according to current data), reported similar findings: opioid naïve patients aged <45 years who received two prescription fills (versus one) or a cumulative dose of 400–799 (versus <120) morphine milligram equivalents in their first month of therapy were 2.3 and 3.0 times as likely to be chronic opioid users, respectively. However, that analysis only examined opioid use in the first month after initiation of opioid therapy to characterize risks for long-term use and did not account for the actual duration of therapy.

The findings in this report are subject to at least five limitations. First, although the cumulative dose of the first episode of opioid use is described, the likelihood of long-term use when the prescriber was titrating the dose was not determined. Rather, the total cumulative dose was calculated, which might have been increasing or decreasing over time. Second, the extent to which chronic opioid use was intentional versus the outgrowth of acute use is not known. Less than 1% of patients in this analysis were prescribed Schedule II long-acting opioids at the outset, so intentional chronic opioid prescribing might be uncommon; however, approximately 10% of patients were prescribed tramadol, which might indicate intentional chronic opioid prescribing. Third, information on pain intensity or duration were not available, and the etiology of pain, which might influence the duration of opioid use, was not considered in the analysis. Fourth, the frequency of prescriptions having certain days’ supplied (e.g., prescriptions with a 7-day supply would be more frequently observed than those with an 11- or 13-day supply) was not considered. The variability in the relationships between days’ supply, the cumulative dose, and duration of first episode and the probability of long-term use could be affected. Finally, prescriptions that were either paid for out-of-pocket or obtained illicitly were not included in the analysis.

Transitions from acute to long-term therapy can begin to occur quickly: the chances of chronic use begin to increase after the third day supplied and rise rapidly thereafter. Consistent with CDC guidelines, treatment of acute pain with opioids should be for the shortest durations possible. Prescribing <7 days (ideally ≤3 days) of medication when initiating opioids could mitigate the chances of unintentional chronic use. When initiating opioids, caution should be exercised when prescribing >1 week of opioids or when authorizing a refill or a second opioid prescription because these actions approximately double the chances of use 1 year later. In addition, prescribers should discuss the long-term plan for pain management with patients for whom they are prescribing either Schedule II long-acting opioids or tramadol.

**Summary**

**What is already known about this topic?**

Based on the CDC Guideline for Prescribing Opioids for Chronic Pain, literature supporting long-term opioid therapy for pain is limited; research suggests an increased risk for harms with long-term opioid use. Early opioid prescribing patterns for opioid-naïve patients have been found to be associated with the likelihood of long-term use.

**What is added by this report?**

In a representative sample of opioid naïve, cancer-free adults who received a prescription for opioid pain relievers, the likelihood of chronic opioid use increased with each additional day of medication supplied starting with the third day, with the sharpest increases in chronic opioid use observed after the fifth and thirty-first day on therapy, a second prescription or refill, 700 morphine milligram equivalents cumulative dose, and an initial 10- or 30-day supply. The highest probability of continued opioid use at 1 and 3 years was observed among patients who started on a long-acting opioid followed by patients who started on tramadol.

**What are the implications for public health practice?**

Awareness among prescribers, pharmacists, and persons managing pharmacy benefits that authorization of a second opioid prescription doubles the risk for opioid use 1 year later might deter overprescribing of opioids. Knowledge that the risks for chronic opioid use increase with each additional day supplied might help clinicians evaluate their initial opioid prescribing decisions and potentially reduce the risk for long-term opioid use. Discussions with patients about the long-term use of opioids to manage pain should occur early in the opioid prescribing process.

## Footnotes

* Morphine milligram equivalents is a conversion factor to convert different opioids into an equivalent dose of morphine. http://www.pdmpassist.org/pdf/BJA_performance_measure_aid_MME_conversion.pdf.

† The six mutually exclusive categories are 1) long-acting: buprenorphine, fentanyl, morphine, oxycodone, oxymorphone, and tapentadol; 2) other Schedule II short-acting: fentanyl, hydromorphone, levorphanol, meperidine, methadone, morphine, oxymorphone and tapentadol; 3) oxycodone short-acting; 4) hydrocodone short-acting; 5) Schedule III–IV and nalbuphine: codeine, dihydrocodeine, butorphanol, nalbuphine, pentazocine and propoxyphene; 6) tramadol.
